# Porous nanoparticles overcome the conventional stiffness-damping tradeoff in polymer nanocomposites

**DOI:** 10.1038/s44431-025-00003-8

**Published:** 2025-10-01

**Authors:** Zhangke Yang, Zhaoxu Meng

**Affiliations:** Department of Mechanical Engineering, Clemson University, Clemson, SC 29634 USA

**Keywords:** Polymers, Nanoscale materials

## Abstract

Nanoparticle-reinforced polymer nanocomposites offer tunable mechanical properties, yet the impact of nanoparticle surface configurations on mechanical and viscoelastic properties remains underexplored. Using coarse-grained molecular dynamics simulations, we investigate how smooth, corrugated, and porous nanoparticles affect the molecular dynamic behaviors of polymer chains, as well as stiffness and damping properties of polymethyl methacrylate (PMMA) nanocomposites. Our results show that all nanoparticle-PMMA systems enhance Young’s and shear moduli, with porous nanoparticles providing the greatest improvements. Stronger interfacial interactions further amplify these effects. A quasi-linear correlation is observed between shear modulus and Debye–Waller factor-based molecular stiffness, with porous nanoparticles exhibiting higher shear moduli due to their unique structural confinement effect. Local molecular stiffness analysis reveals pronounced dynamic heterogeneity, leading to heterogeneous strain distributions under shear deformation. Small amplitude oscillatory tests demonstrate simultaneous enhancements in stiffness and damping, overcoming the conventional tradeoff. These findings highlight the potential of tailoring nanoparticle surface configurations for designing polymer nanocomposites with superior mechanical performance.

## Introduction

Polymers are widely used as the matrix phase for nanocomposites^1–3^, exemplified by systems such as graphene-polymethyl methacrylate (PMMA)^4–6^, clay-epoxy^7^, and clay-polyurethane^8,9^ nanocomposites. While the polymer matrix defines the baseline mechanical properties, incorporating nanofillers can significantly modify these properties due to their intrinsic characteristics and complex interactions with the polymer matrix. Consequently, polymer nanocomposites often exhibit much enhanced mechanical properties compared to pure polymer, with optimized improvements achieved by appropriately selecting and arranging the nanofillers. Nanofillers vary in configuration based on their dimensions, including two-dimensional (2D) nanosheets^10^, one-dimensional (1D) nanofibers/nanotubes^11^, and zero-dimensional (0D) nanoparticles^12^. Prior studies have shown that graphene, with its 2D sheet structure and high surface-to-volume ratio, is an exceptional nanofiller for polymer matrices^6,13,14^. In our previous work, layered graphene-PMMA nanocomposite films demonstrated remarkable impact energy dissipation, a property largely attributed to the unique arrangement of the graphene layers^4^. These films also exhibit significantly increased elastic moduli, a result of the nanoconfinement effect of graphene sheets on PMMA chains^5,15,16^. Moreover, carbon nanotubes—including multiwalled carbon nanotubes (MWCNTs), which are 1D derivatives of graphene formed by wrapping one or more graphene layers—have also been observed to impart notable mechanical reinforcement in nanocomposites, as evidenced by MWCNT–poly(styrene–butyl acrylate) latex systems^17^.

Similarly, nanoparticle-polymer nanocomposites have attracted considerable attention for applications ranging from food packaging^18,19^ and cancer therapy^20^ to materials with tailored optical and magnetic properties^21–23^. Unlike nanosheets or nanofibers, nanoparticles have been explored earlier as nanofillers due to their straightforward synthesis and ease of dispersion within polymer matrices. For example, silver nanoparticle-reinforced polymer nanocomposites can be produced via physical methods—such as roll-to-roll^24^, dip-coating^25^, and compression^26^ techniques—or through chemical approaches like in situ^27^ and ex situ^28^ polymerization processes. Studies have demonstrated a clear mechanical reinforcement effect in silica-polymer nanocomposites, particularly at higher temperatures, with reinforcement effects varying with nanoparticle sizes and polymer types^29^. Notably, silica–epoxy and rubber–epoxy nanocomposites have exhibited significantly increased fracture toughness in compact tension tests^12^. In one study, incorporating less than 10 wt% of 12-nm spherical silica nanoparticles in an epoxy matrix resulted in a 25% increase in tensile modulus and a 30% improvement in fracture toughness. Although higher nanoparticle loadings may introduce tradeoffs among modulus, strength, and fracture toughness, the overall mechanical enhancements provided by silica nanoparticles remain evident^30^.

It comes as no surprise that 0D nanoparticles can significantly enhance the mechanical properties of polymer nanocomposites, given that many biomaterials rely on such nanoscale reinforcement mechanisms to achieve tunable performance. One prominent example is the bone, which combines soft, extensible collagen fibrils with hard mineral phases arranged in hierarchical structures to attain exceptional mechanical properties. Interestingly, many natural bio-nanocomposites incorporate irregular-shaped or porous nanoparticles to reinforce biopolymers. For instance, the dactyl club of mantis shrimp features a coating layer composed of porous hydroxyapatite nanoparticles embedded in a biopolymer matrix, and it has been found that this unique configuration contributes to its remarkable impact resistance^31^. Moreover, in bone, the mineral particles are typically arranged as thin plates aligned parallel to the collagen fibrils, while the plates exhibit irregular shapes and rough surfaces^32,33^. Although hydroxyapatite crystals are brittle in isolation, the resulting nanocomposite of hydroxyapatite and collagen exhibits impressive toughness and resistance to crack propagation^34^. Similarly, in nacre, aragonite platelets display highly uneven surfaces with nano-asperities, further enhancing the material’s mechanical resilience^35,36^.

Recent advances in manufacturing and processing techniques have greatly broadened the range of nanoparticles that can be synthesized, offering versatile control over their chemistry, geometry, and surface characteristics. Although microbial synthesis methods are still limited by slower production rates, the rich biodiversity of microbes and continuous improvements in techniques make this approach one of the most promising for metal nanoparticle synthesis^37^. Notably, most synthesized nanoparticles tend to exhibit irregular shapes or rough surfaces. Additionally, templating approaches have been widely employed to produce porous metal nanoparticles^38–40^. For instance, Chen et al. used CaCO_3_ as the inorganic template to synthesize spherical porous hollow silica nanoparticles with good dispersion for drug carrier applications^41^. Similarly, employing P-123 copolymers as a template in an aqueous solution enabled the fabrication of porous zinc oxide (ZnO) nanoparticles from surface-modified colloidal ZnO nanocrystallites^42^. Replacement reaction has also been utilized to fabricate metal nanostructures with hollow interiors^43^ and to achieve shape-controlled synthesis of metal nanoparticles^44^. Additionally, Teng et al. employed a self-organization process to synthesize nearly monodispersed porous platinum nanoparticles^45^.

Capabilities in versatile control over nanoparticle geometry and surface characteristics prompt researchers to investigate how these characteristics impact the mechanical performance of nanocomposites. Such insights are poised to integrate innovative surface engineering strategies into the design of next-generation polymer nanocomposites. Naturally, the configuration of nanoparticle surfaces is expected to play a pivotal role in determining mechanical properties. In particular, nanoparticles with corrugated or porous surfaces have significantly larger surface-to-volume ratios compared to regular-shaped nanoparticles, which can lead to enhanced interfacial bonding and, as a result, more pronounced improvements in mechanical performance.

Despite this compelling rationale and the widespread occurrence of such configurations in nature materials^31,46,47^, direct evidence linking surface-corrugated or porous nanoparticles to improved mechanical properties remains scarce. The primary challenges stem from experimental difficulties in isolating the effects of nanoparticle surface features from other contributing factors, compounded by substantial random variances inherent in material systems. Moreover, traditional simulation techniques have struggled to capture the multilevel structure of nanocomposites—ranging from individual polymer chains and specific nanoparticle surface details to the holistic behavior of the nanocomposite system. Consequently, the specific influence of nanoparticle surface configurations on mechanical property reinforcement and the underlying mechanisms driving these effects have remained largely elusive.

**Fig. 1:**
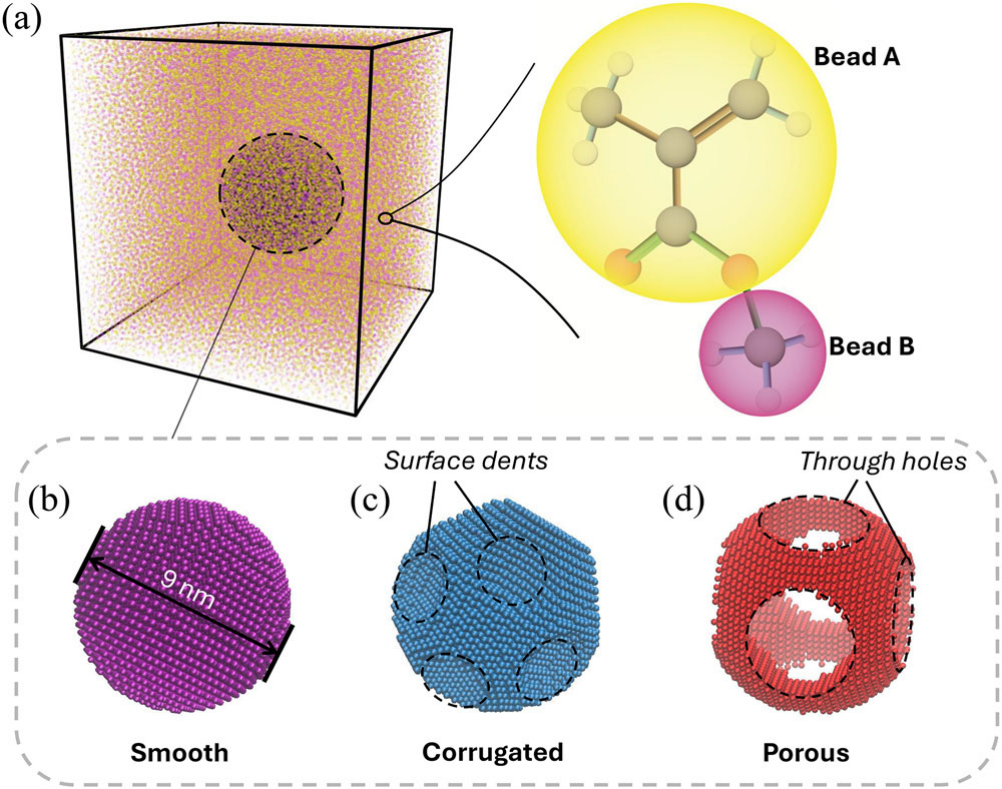
Computational model setup. **a** Representative nanocomposite model showing the coarse-grained representation of a PMMA monomer and configurations of **b** smooth, **c** corrugated, and **d** porous nanoparticles.

To overcome these challenges, we employ coarse-grained (CG) molecular dynamics (MD) simulations to systematically investigate the roles of nanoparticle surface configurations in enhancing the mechanical properties of polymer nanocomposites. Our models incorporate both detailed nanoparticle surface features and molecular-level representations of polymer chains (Fig. 1), allowing us to probe overall mechanical property enhancements and the regional, molecular-level thermodynamical behaviors that drive these enhancements. We examine three representative nanoparticle types: smooth spherical nanoparticles with uniform surfaces (smooth), spherical nanoparticles with corrugated surfaces (corrugated), and spherical porous nanoparticles (porous), as illustrated in Fig. 1. Mechanical properties under tensile and shear deformations are characterized, and we further assess the molecular stiffness and dynamic heterogeneity of the polymer chains to elucidate the dependence on nanoparticle surface configurations. Additionally, our analysis of viscoelastic properties reveals that porous nanoparticles offer unique advantages, providing valuable insights into the design of advanced polymer nanocomposites.

## Results

### Comparison of elastic moduli of different nanocomposites

**Fig. 2:**
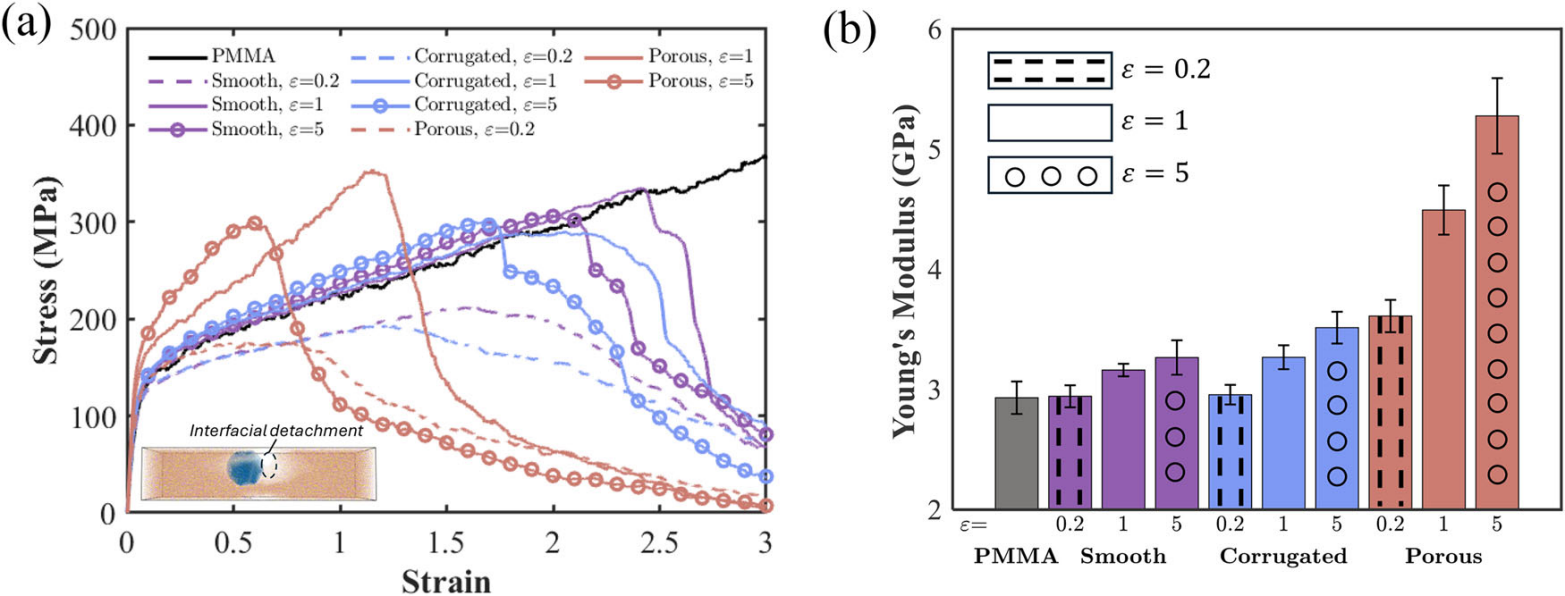
Uniaxial tensile results. **a** Representative tensile stress-strain curves for pure PMMA and nanoparticle-PMMA nanocomposites reinforced with smooth, corrugated, and porous nanoparticles. The inset highlights interfacial detachment around a corrugated nanoparticle at $\varepsilon$ = 0.2 ${{\rm{kcal}}}/{{\rm{mol}}}$. **b** Young’s moduli of PMMA and the nanocomposites extracted from the tensile stress-strain curves. Error bars represent the standard deviations from five independent simulations with different initial configurations.

Figure 2 presents the tensile test results for the three types of nanoparticle-PMMA nanocomposites compared with pure PMMA. For each nanocomposite, we investigate three different $\varepsilon$ values ($\mathrm{0.2},\,{1},\,{{\rm{and}}}$ $5\,{{\rm{kcal}}}/{{\rm{mol}}}$), where a higher $\varepsilon$ corresponds to stronger interfacial interactions^4,48^. Figure 2a displays representative tensile stress-strain curves for pure PMMA and the three types of nanocomposites across these $\varepsilon$ values. In each case, the stress-strain curve exhibits an initial linear elastic region, indicating that all three types of nanocomposites maintain linear elastic behavior under small strains. Young’s modulus is determined by calculating the slope of this linear portion of the corresponding tensile stress-strain curve^49^, specifically using the strain range of 0 to 0.015.

Figure 2b offers a comprehensive comparison of Young’s moduli for pure PMMA and all three types of nanoparticle-PMMA nanocomposites at different $\varepsilon$ values, with error bars representing the standard deviations from five independent simulations using distinct initial configurations. Overall, the porous nanoparticle systems demonstrate the highest enhancement in Young’s modulus, followed by the corrugated and smooth systems. Moreover, increasing $\varepsilon$ significantly enhances Young’s moduli of the nanocomposites, with the porous cases showing the greatest sensitivity to increased interactions between nanoparticles and polymer chains. We attribute this pronounced enhancement and sensitivity in the porous systems to a unique structural confinement effect, whereby polymer chains are confined within the through-holes of the porous nanoparticles.

At weak interfacial interaction level ($\varepsilon =0.2\,{{\rm{kcal}}}/{{\rm{mol}}}$), both the smooth and corrugated nanoparticle-reinforced nanocomposites offer no discernible improvement in Young’s modulus over pure PMMA, whereas the porous nanoparticle-reinforced nanocomposite exhibits a marked enhancement (over 20%). This distinct behavior highlights the unique structural confinement effect of porous nanoparticles, which reinforce the polymer matrix even when interfacial interactions are weak. In contrast, smooth and corrugated nanoparticles reinforce the PMMA matrix by relying solely on interfacial interactions. When the interactions between nanoparticles and PMMA are weak, nanoparticles show negligible reinforcement effects or even have a detrimental effect on the mechanical properties of the polymer phase by creating free surfaces in the polymer^50,51^. As $\varepsilon$ increases and interfacial interaction strengthens, both smooth and corrugated systems begin to exhibit an enhancement effect on Young’s modulus. The corrugated case shows a slightly greater increase in Young’s modulus compared to the Smooth case, attributed to the marginally larger surface area of the corrugated nanoparticles relative to the smooth nanoparticles.

Furthermore, the structural confinement provided by porous nanoparticles restricts PMMA chain mobility, reducing plastic deformation and leading to earlier strain localization and failure, as evidenced by the earlier drop in the porous systems. Similarly, the addition of smooth and corrugated nanoparticles reduces the plastic deformation of PMMA chains. This can be explained by the similar strain localization caused by nanoconfinement that hinders the overall plastic deformation of PMMA. This nanoconfinement effect tends to be more significant with higher $\varepsilon$. At low $\varepsilon$ (0.2 ${{\rm{kcal}}}/{{\rm{mol}}}$), however, interfacial detachment (as shown in the inset of Fig. 2a) occurs more easily, leading to a significant reduction in load-bearing capabilities, as indicated by the dashed lines in Fig. 2a. The interplay between interfacial detachment and strain localization with increasing $\varepsilon$ leads to the observation that a moderate $\varepsilon$ of 1 ${{\rm{kcal}}}/{{\rm{mol}}}$ consistently leads to larger strains before the stress drop, compared to both lower and higher $\varepsilon$ values.

### Shear modulus, molecular stiffness, and shear deformation analysis

**Fig. 3:**
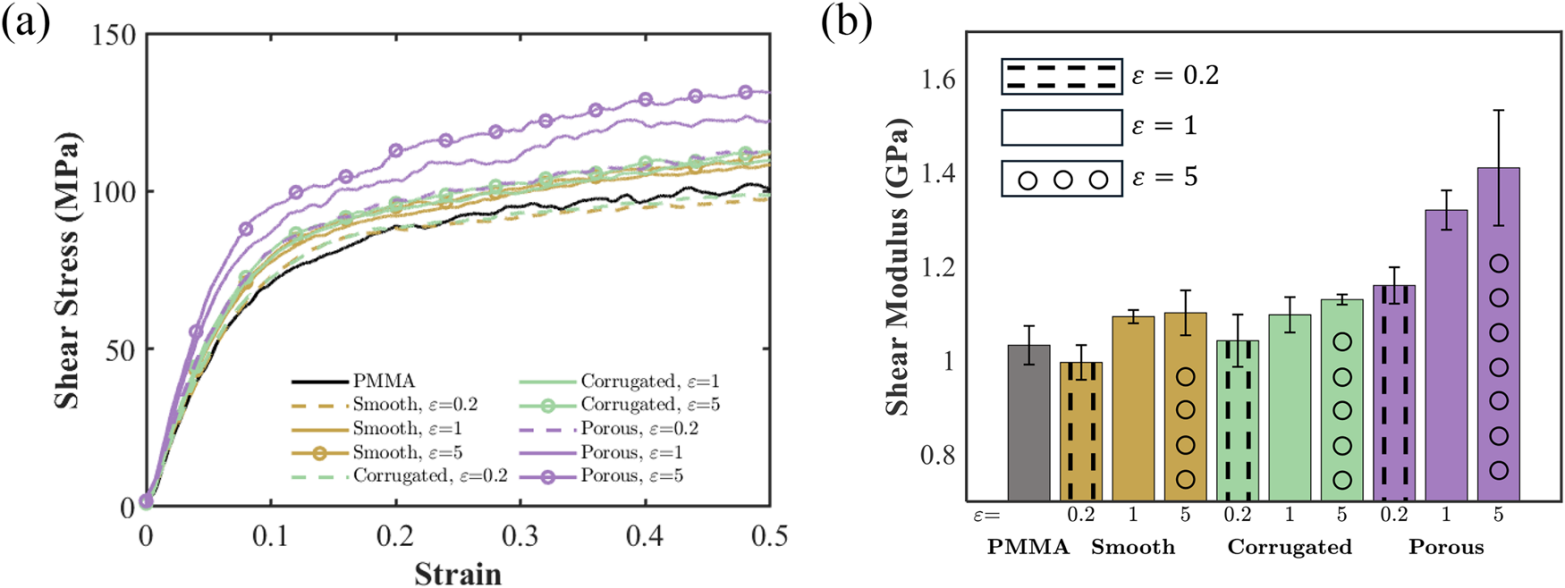
Shear test results. **a** Representative shear stress-strain curves for pure PMMA and nanoparticle-PMMA nanocomposites. **b** Corresponding shear moduli extracted from the shear stress-strain curves. Error bars represent the standard deviations obtained from five independent simulations with different initial configurations.

We examine the nanocomposites’ mechanical behaviors under shear deformations depending on nanoparticle configurations. Figure 3 presents the stress-strain curves and shear moduli results for the three types of nanoparticle-PMMA nanocomposites, compared against pure PMMA. Similar shear stress-strain curves are observed, characterized by an initial quasi-linear elastic region, a nonlinear transition phase, and a subsequent plateau. The shear moduli of the materials are determined by calculating the slopes of the initial quasi-linear elastic regions of the shear stress-strain curves, and the results are shown in Fig. 3b.

Notable differences are observed between the trends of the shear moduli and Young’s moduli. For instance, the enhancement of the shear moduli is less sensitive to $\varepsilon$ than that of Young’s moduli, particularly for the smooth and corrugated nanoparticle-PMMA nanocomposites. Furthermore, the shear modulus of the smooth case with $\varepsilon =0.2\,{{\rm{kcal}}}/{{\rm{mol}}}$ is noticeably lower than that of pure PMMA. This phenomenon is attributed to the low shear resistance of the weak and smooth interfaces, giving rise to easier sliding of PMMA chains under shear deformation. Nevertheless, the overall trend in shear moduli shares many similarities with Young’s modulus. Specifically, porous cases exhibit the greatest improvement in shear moduli, followed by corrugated and smooth Cases. Additionally, higher $\varepsilon$ leads to greater shear modulus for each nanoparticle configuration.

Next, we correlate the shear modulus results with the dynamics of the systems under equilibrium state by characterizing the mean-field molecular stiffness of the system, which is inversely proportional to the Debye–Waller factor (DWF), ($\langle {u}^{2}\rangle$). This inverse relationship arises from the observation that the high-frequency shear modulus is inversely related to the MSD of particles that are transiently localized on time scales longer than the average particle collision time but shorter than the structural relaxation time^52^. Following previous validations^53^, we define the mean-field molecular stiffness as $1/\langle {u}^{2}\rangle$. In this study, $\langle {u}^{2}\rangle$ is obtained from the MSD ($\langle {r}^{2}(t)\rangle$) of all polymer beads at $t=4\,{{\rm{ps}}}$, which corresponds to the characteristic caging time for the PMMA CG model^54^.

**Fig. 4:**
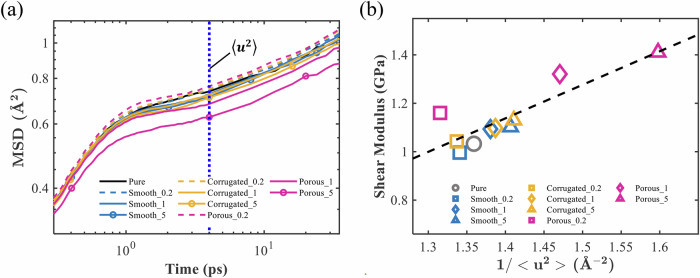
Mean-field molecular stiffness characterization and correlation with shear modulus. **a** MSDs of pure PMMA and nanoparticle-PMMA nanocomposites at different $\varepsilon$ values. The $\langle {u}^{2}\rangle$ value for each system, calculated at $t=4{{\rm{ps}}}$, serves as an inverse indicator of the mean-field molecular stiffness. **b** Shear moduli vs. $1/\langle {u}^{2}\rangle$ for pure PMMA and the nanocomposites. The dashed line indicates the least-squares linear fit.

The evolution of the MSDs (〈*r*^2^ (*t*)〉) of the three types of nanoparticle-PMMA nanocomposites and pure PMMA is shown in Fig. 4a. It can be observed that the caging effect is almost nonexistent on time scales shorter than a picosecond. As the system enters the caging regime, where each particle becomes confined by its surrounding particles, the MSDs of the three types of nanocomposites and PMMA exhibit nontrivial differences. Specifically, porous cases show the lowest MSDs, followed by the corrugated, smooth, and finally pure PMMA. Furthermore, the higher the $\varepsilon$, the smaller the MSD, consistent with previous studies^53^.

The mean-field molecular stiffness, represented by $1/\langle {u}^{2}\rangle$, shows inversed dependence on the type of nanoparticles and $\varepsilon$ values. Figure 4b illustrates the quasi-linear correlation between shear moduli and molecular stiffness results, as indicated by the dashed least-squares fit to all data points. Notably, the relationship between the shear moduli and $1/\langle {u}^{2}\rangle$ for the porous nanoparticle-PMMA nanocomposites deviates from this overall trend: at equivalent levels of molecular stiffness, they exhibit higher shear moduli compared to other systems. We attribute this deviation to the unique structural confinement effect of the porous nanoparticles, which markedly influences the shear moduli under nonequilibrium shear deformations.

**Fig. 5:**
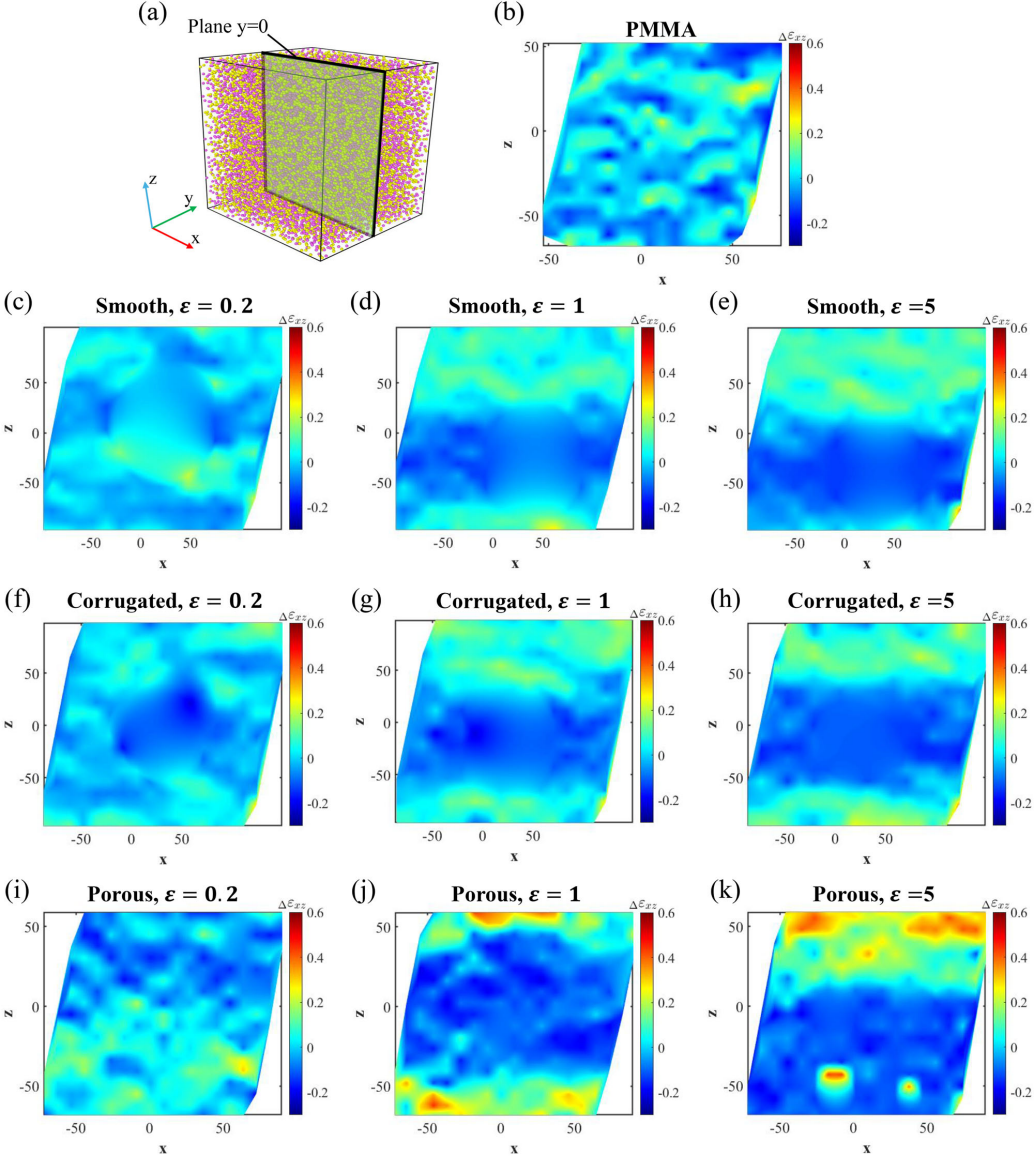
Characterization of heterogeneous shear strain distribution. **a** Schematic of the sliced plane at $y=0$ used for analysis. Contour maps show the local shear strain deviation, ${\Delta \varepsilon }_{{xz}}$, defined as the difference between local shear strain (${\varepsilon }_{{xz\_l}}$) and global shear strain (${\varepsilon }_{{xz\_g}}=0.22$). **b** Pure PMMA, **c**–**e** nanocomposites with smooth nanoparticle, **f**–**h** nanocomposites with corrugated nanoparticle, and **i**–**k** nanocomposites with porous nanoparticle. Interpolation and smoothing have been applied in regions where no PMMA beads are present.

To elucidate the deformation mechanisms of nanoparticle-PMMA nanocomposites with varying nanoparticle configurations under shear loading, we analyze shear strain contours that depict the deviation $({\Delta \varepsilon }_{{xz}})$ between the local shear strain (${\varepsilon }_{{xz\_l}}$) and the externally applied global shear strain (${\varepsilon }_{{xz\_g}}$) on the shear plane at $y=0$ (Fig. 5a). Without losing generality, we select ${\varepsilon }_{x{z}_{g}}=0.22$ for illustration in Fig. 5. We note that only ${\Delta \varepsilon }_{{xz}}$ of the PMMA matrix is plotted since the nanoparticles are treated as rigid bodies in this study, and the nanoparticles may shift slightly from the center during shear. Furthermore, interpolation and smoothing are applied to the contours for areas on the plane *y* = 0 where no PMMA bead is present.

The pure PMMA exhibits a relatively homogenous shear strain with only minor fluctuations in ${\Delta \varepsilon }_{{xz}}$ values, as shown in Fig. 5b. In contrast, Δ*ε*_*xz*_ contours of the nanocomposites reveal pronounced heterogeneity, with distinct regions exhibiting either higher or lower local strains than ${\varepsilon }_{{xz\_g}}$. This effect is particularly evident in the porous nanoparticle case with high $\varepsilon$, i.e., strong interfacial interaction. Moreover, the degree of heterogeneity in shear deformation increases with increasing $\varepsilon$. Such a heterogeneous shear strain distribution suggests that the local modulus within the nanocomposites varies spatially—a characteristic reminiscent of the dynamic heterogeneity observed in glass-forming polymer materials, which we analyze further next.

### Dynamic heterogeneity of the nanoparticle-PMMA nanocomposites

**Fig. 6:**
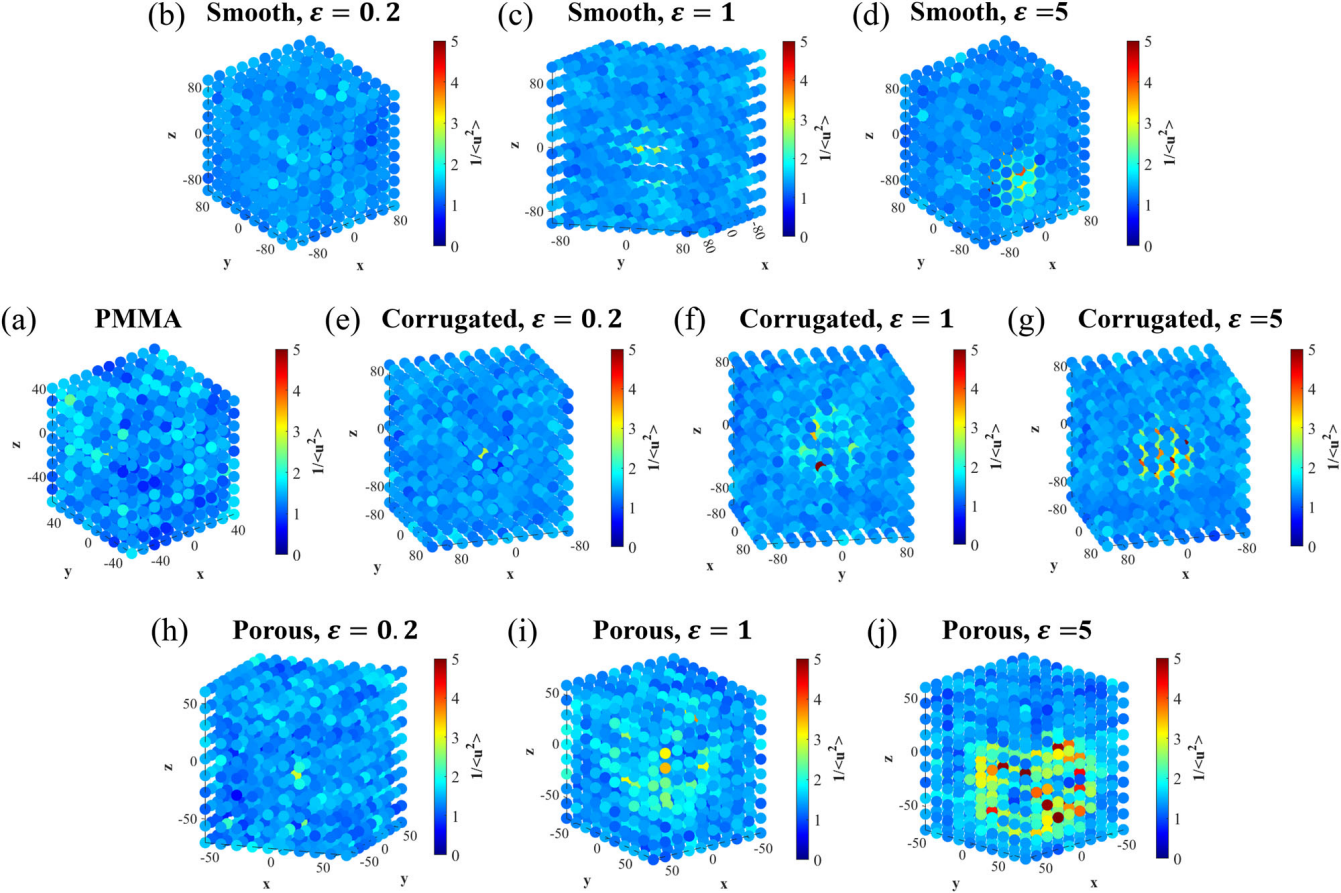
Dynamic heterogeneity manifested by local molecular stiffness. Contour maps of the local molecular stiffness, $1/\langle {u}^{2}\rangle$, for **a** pure PMMA and nanocomposites with **b**–**d** smooth, **e**–**g** corrugated, and **h**–**j** porous nanoparticles at different *ε* values.

Motivated by the observed heterogeneous deformation, we further explore and compare the dynamic heterogeneity of the nanocomposites and PMMA. To address this, we divide the computational domain into 1000 cells ($10\times 10\times 10$ grid) and evaluate the local molecular stiffness ($1/\langle {u}^{2}\rangle$) as the reciprocal of the MSD of beads in each cell at $t=4{{\rm{ps}}}$. Figure 6 presents contour maps depicting the local molecular stiffness for PMMA and the three types of nanocomposites with different $\varepsilon$ values.

For pure PMMA (Fig. 6a), local molecular stiffness is uniform with only slight thermal fluctuations^55^, reflecting its homogeneous mechanical properties. At $\varepsilon =0.2\,{{\rm{kcal}}}/{{\rm{mol}}}$, all three types of nanocomposites display relatively uniform local molecular stiffness distributions, indicating low dynamic heterogeneity. As the interfacial interaction increases to $\varepsilon =1\,{{\rm{kcal}}}/{{\rm{mol}}}$, the PMMA chains in the vicinity of the nanoparticle exhibit noticeably higher local molecular stiffness than the rest of the matrix. At $\varepsilon =5\,{{\rm{kcal}}}/{{\rm{mol}}}$, distinct regions of elevated local molecular stiffness become evident near the nanoparticles in all three nanocomposites. In the smooth and corrugated nanoparticle-PMMA nanocomposites, the regions of enhanced local molecular stiffness form a shell-like layer around each nanoparticle. In contrast, for the porous nanoparticle-PMMA nanocomposite, the region of enhanced local molecular stiffness extends from the exterior to the interior of the porous nanoparticle, indicating a strong structural confinement effect for the PMMA chains penetrating the through holes of porous nanoparticles.

The dynamic heterogeneity observed in the nanoparticle-PMMA nanocomposites explains the nonuniform shear strain distributions shown in Fig. 5. Specifically, regions exhibiting enhanced local molecular stiffness experience less deformation than areas without such enhancement.

### Viscoelastic properties of the nanoparticle-PMMA nanocomposites

**Fig. 7:**
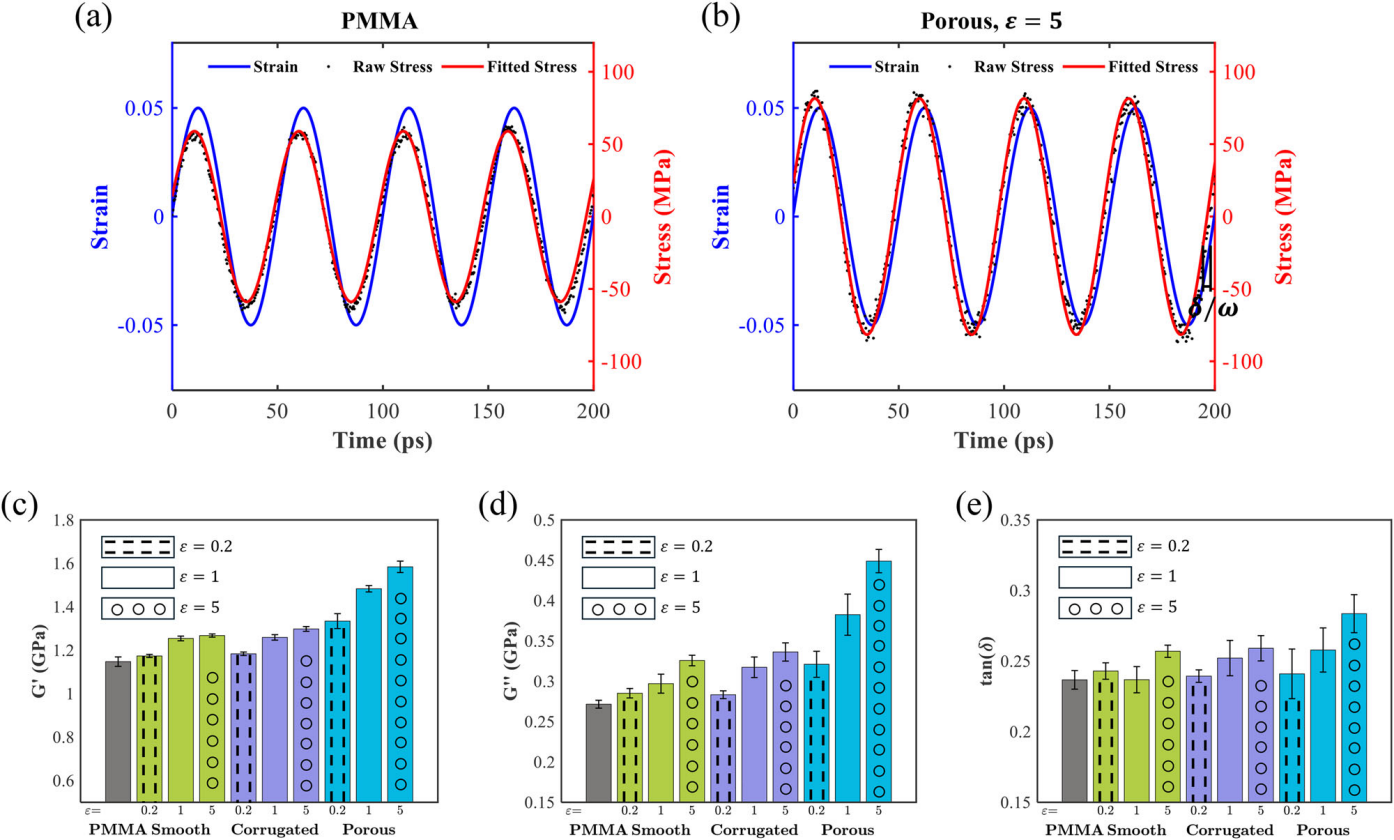
SAOS results. Representative strain input and corresponding fitted stress output curves under SAOS tests for **a** pure PMMA and **b** the porous nanoparticle-PMMA nanocomposite with *ε* *=* 5 kcal/mol. Comparison of **c** storage moduli ($G^{\prime}$), **d** loss moduli ($G^{{\prime} {\prime}}$), and **e** loss tangent ($\tan (\delta )$) for pure PMMA and nanocomposites reinforced with smooth, corrugated, and porous nanoparticles at different *ε* values. Error bars represent the standard deviations calculated from five independent simulations with different initial configurations.

We then utilize small amplitude oscillatory shear (SAOS) tests^56^ to examine and compare the viscoelastic properties of the three types of nanocomposites against those of pure PMMA. The viscoelastic behavior of polymers is predominantly governed by the sliding motion of polymer chains^57,58^. As demonstrated earlier, the incorporation of nanoparticles significantly alters chain dynamics and local molecular stiffness, with specific effects depending on the surface configurations of the nanoparticles and their interactions with PMMA. This observation raises the question of how these unique factors influence the viscoelastic response of the nanocomposites. Figure 7a, b displays representative stress-strain curves for pure PMMA and for a nanocomposite with porous nanoparticle and interfacial interaction of $\varepsilon =5\,{{\rm{kcal}}}/{{\rm{mol}}}$. Additionally, Fig. 7c–e compares the dynamic moduli ($G^{\prime}$, $G^{{\prime} {\prime}}$, and $\tan (\delta )$) across different cases.

The blue curves in Fig. 7a, b illustrate the sinusoidal strain input applied during the oscillatory shear, characterized by an amplitude of 0.05 and a period of 50 ps, respectively. Superimposed on these curves, clusters of the black points depict the resulting stress histories, which follow a similar sinusoidal pattern but exhibit a phase shift ($\delta$). This $\delta$ is a crucial indicator of the material’s damping capability: a zero-phase shift $(\tan (\delta )=0$) implies a purely elastic, immediate stress response to strain, while $\delta =\frac{\pi }{2}$ signifies a purely viscous behavior. When the phase shift lies between the two extremes, i.e., $0 < \delta < \frac{\pi }{2}$, the material is classified as viscoelastic^59^, with a higher tan $(\delta )$ indicating a more viscous and less elastic response.

The red lines in Fig. 7a, b represent the fitted sinusoidal stress curves. Notably, the fluctuations of the raw stress data (black points) around the fitted curves are more pronounced for the porous nanoparticle-PMMA nanocomposite compared to pure PMMA. This increased fluctuation is attributed to the presence of nanoparticles, which enhance dynamic heterogeneity and result in a less smooth deformation response. Nonetheless, the overall sinusoidal trend of the stress data confirms that a viscoelastic model is appropriate for describing the behavior of these nanocomposites.

Figure 7c–e compares the dynamic moduli—$G^{\prime}$, $G^{{\prime} {\prime}}$, and $\tan (\delta )$—across the three nanoparticle–PMMA nanocomposites and pure PMMA. All three nanocomposite types exhibit an increase in $G^{\prime}$ compared to pure PMMA, with the porous case providing the greatest enhancement, followed by nearly identical improvements for corrugated and smooth cases. Moreover, for each nanoparticle type, the enhancement of $G^{\prime}$ becomes more pronounced with $\varepsilon$. Notably, the porous nanoparticle-PMMA nanocomposite at $\varepsilon =0.2\,{{\rm{kcal}}}/{{\rm{mol}}}$ shows a higher $G^{\prime}$ than the other two systems at $\varepsilon =5\,{{\rm{kcal}}}/{{\rm{mol}}}$. This significant advantage is primarily attributed to the structural confinement effect imparted by the porous nanoparticles.

For many engineering materials, achieving both high stiffness and effective energy dissipation (damping) is challenging, as these properties are often mutually exclusive^60^. Remarkably, our results indicate that porous nanoparticles enhance not only $G^{\prime}$, but also $G^{{\prime} {\prime}}$ and tan $(\delta )$. As shown in Fig. 7d, e, porous nanoparticles are generally advantageous in improving the damping capability of the nanocomposites. The results also show that the three types of nanoparticle-PMMA nanocomposites typically exhibit higher tan (*δ*) values than pure PMMA, and these values generally increase with $\varepsilon$. This is a significant finding, as both stiffness and damping enhancements have been achieved through the unique surface configurations of the nanoparticles, particularly in the porous nanoparticle-PMMA nanocomposites. We attribute these improvements primarily to the enhanced dynamic heterogeneity induced by the nanoparticles within the PMMA matrix. Previous studies have shown that leveraging bioinspired structures can overcome the traditional performance tradeoff between stiffness and damping^61–64^. In this study, we extend this concept by demonstrating that tailoring nanoparticle surface features in polymer nanocomposites offers a promising strategy to break this performance tradeoff.

As discussed earlier, nanoparticle features such as corrugated surfaces, porosity, and enhanced interfacial interactions induce pronounced dynamic heterogeneity, which contributes to improved stiffness in the nanocomposites. The results herein extend this understanding by demonstrating that dynamic heterogeneity also plays a crucial role in enhancing damping. The damping of polymers arises primarily from molecular chain motions that generate internal friction, with chain sliding being a significant contributor^56,65^. Modifying the surface configurations of nanoparticles alters the uniformity of local shear strain, as shown in Fig. 5, leading to variations in the extent of internal chain sliding during oscillatory deformation.

**Fig. 8:**
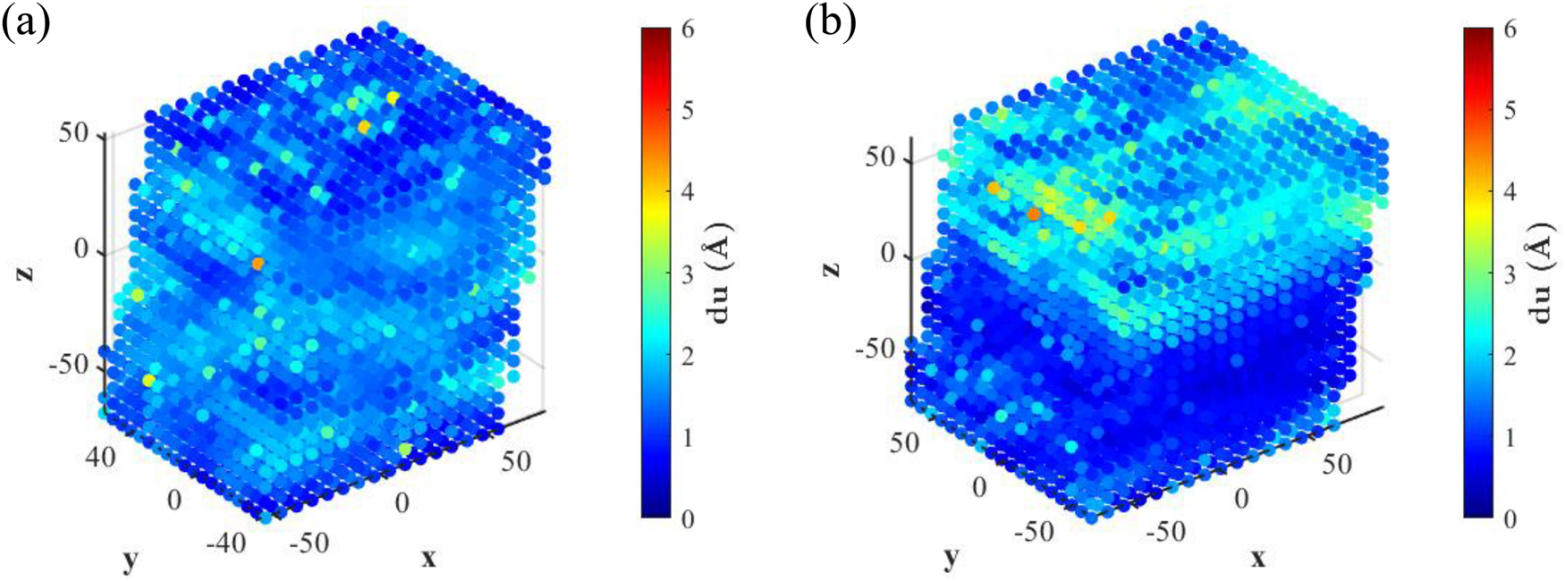
Chain sliding behavior characterized by *du.* Contour maps of ${du}$ for **a** pure PMMA and **b** the porous nanoparticle-PMMA nanocomposite (*ε* *=* 5 kcal/mol) at a global shear strain of 0.22.

To further demonstrate that the enhanced $G^{{\prime} {\prime}}$ and $\tan (\delta )$ in the nanocomposites arise from internal chain sliding under oscillatory shear, we introduce the concept of differential displacement (${du}$), defined as:

1 $${du}(i)=\frac{1}{N}\mathop{\sum }\limits_{j=1}^{N}\left|\vec{{u}_{i}}-\vec{{u}_{j}}\right|$$

where ${du}(i)$ is the differential displacement of bead $i$, $N$ is the total number of beads within 15 $\mathring{\rm A}$ of bead $i$, and $\vec{{u}_{i}}$ and $\vec{{u}_{j}}$ denote the displacements of bead $i$ and $j$ from the moment when the shear strain is 0. The metric effectively quantifies the extent of local sliding, particularly when bead displacements are predominantly in the same direction under significant shear strain. Figure 8a, b displays the contours of ${du}$ for pure PMMA and the porous nanoparticle-PMMA nanocomposite with $\varepsilon =5\,{{\rm{kcal}}}/{{\rm{mol}}}$ at a shear strain of 0.22, respectively. In pure PMMA, chain sliding is relatively uniform, whereas the porous nanocomposite exhibits significantly higher ${du}$ in localized regions, corresponding closely with the high-strain areas observed in Fig. 5. Additionally, the interfacial sliding observed at the nanoparticle-PMMA interfaces in Fig. 8b further contributes to the enhanced damping behavior^66^.

## Discussion

In this study, we investigated the effect of nanoparticle surface configurations on the mechanical properties of nanoparticle-PMMA nanocomposites using CG MD simulations. Representative RVE models were constructed for pure PMMA and for nanocomposites reinforced with smooth, corrugated, and porous nanoparticles. Uniaxial tensile and shear tests revealed that incorporating nanoparticles significantly enhances both Young’s and shear moduli compared to pure PMMA, with porous nanoparticles yielding the highest stiffness improvements, followed by corrugated and smooth nanoparticles. Additionally, increasing the PMMA-nanoparticle interfacial interaction further boosts these moduli.

At the RVE scale, our analysis revealed a quasi-linear relationship between shear modulus and mean-field molecular stiffness, quantified by the overall MSD. Notably, nanocomposites with porous nanoparticles exhibit higher shear moduli at equivalent levels of mean-field molecular stiffness, a phenomenon attributed to the unique structural confinement effect induced by the porous configuration. While the current study focused on a single porosity level (28%), we anticipate that both porosity and pore geometry could play critical roles in modulating the mechanical properties of the nanocomposites through interfacial interactions and structural confinement effects. These effects will be systematically explored in our future work.

Our simulations further revealed pronounced nonuniform shear strain distributions within the nanocomposites, with the greatest strain heterogeneity observed in porous nanoparticle-reinforced systems at the highest $\varepsilon$. Additionally, contour maps of local molecular stiffness, derived from local MSD measurements, demonstrated that the nanocomposites—particularly those reinforced with porous nanoparticles—exhibit significantly greater dynamic heterogeneity than pure PMMA. This effect is further amplified by stronger PMMA-nanoparticle interfacial interactions.

Our SAOS tests revealed substantial simultaneous enhancements in stiffness and damping—two properties traditionally considered mutually exclusive in engineering materials. Furthermore, visualization of differential displacement revealed that the heterogeneous strain distributions promote PMMA chain sliding, which directly contributes to the enhanced damping performance of the nanocomposites. These findings indicate that the increased dynamic heterogeneity and unique structural confinement simultaneously enhance local molecular stiffness and damping properties.

Our results are consistent with previous experimental findings. For example, Zaragoza et al.^67^ embedded silica nanoparticles into hydrogels during in situ polymerization and reported up to a threefold enhancement in Young’s modulus at 5% $w/v$ nanoparticle loading. Notably, their study demonstrated simultaneous enhancements in both $G^{\prime}$ and $\tan \delta$, thereby overcoming the conventional stiffness–damping tradeoff. Similarly, Wang et al.^68^ developed polymer-grafted silica nanoparticles with various grafted chains on both solid and porous silica nanoparticles. The resulting bulk films exhibited improved mechanical properties and enhanced CO_2_ permeability. In comparison, our study demonstrated a comparable enhancement in both stiffness and damping performance. Specifically, nanocomposites reinforced with porous nanoparticles and strong interfacial interactions ($\varepsilon =5\,{{\rm{kcal}}}/{{\rm{mol}}}$) exhibited a 180% increase in Young’s modulus and 140% increase in shear modulus. Unlike prior studies that focused on experimental performance metrics, our work offers molecular-level mechanistic insights into how nanoparticle surface morphology and interfacial interaction strength synergistically affect nanocomposite performance.

Our findings align with previous research on the impact-resistant coating of the dactyl club of mantis shrimps, which consists of porous hydroxyapatite nanoparticles interpenetrated within an organic matrix^31^. This unique bi-continuous structure enables the mantis shrimp to achieve an exceptional combination of stiffness and damping, effectively localizing damage and dissipating impact energy under extreme high-strain-rate conditions^31^. Similarly, our study demonstrates that porous nanoparticles within polymer nanocomposites introduce a unique structural confinement effect and heightened dynamic heterogeneity, leading to the simultaneous enhancement of stiffness and damping—two properties traditionally considered mutually exclusive in engineered materials. By elucidating the fundamental mechanisms underlying this dual enhancement, our findings provide valuable insights into the role of nanoparticle surface configurations in optimizing mechanical performance, paving the way for bioinspired designs of advanced polymer nanocomposites.

Overall, our findings underscore the pivotal role of nanoparticle surface configurations in simultaneously tailoring stiffness and damping in polymer nanocomposites, providing valuable guidelines for the design of advanced materials with superior mechanical performance.

## Methods

### CG models of nanocomposites with specific nanoparticle surface configurations

In this study, we use a representative volume element (RVE) consisting of polymer chains surrounding a nanoparticle with different configurations within a cubic simulation box, as shown in Fig. 1a. Periodic boundary conditions are applied in all three directions of the simulation box, effectively replicating the system infinitely. It is worth noting that this RVE configuration assumes a uniform distribution of nanoparticles throughout the polymer matrix.

PMMA, the polymer considered in this study, has been widely used in various engineering applications^69–72^. We employ a well-established CG representation in which each monomer consists of a backbone group (C_4_O_2_H_5_) mapped to bead A and a side-chain group (CH_3_) mapped to bead B^73^. The bulk PMMA in the CG configuration surrounding the nanoparticle is shown in Fig. 1a, with yellow and purple beads representing types A and B, respectively. Bead A is centered at the quaternary carbon atom of the methacrylate group, while bead B is positioned at the side-chain carbon atom. Non-bonded interactions among beads are modeled using Lennard-Jones (LJ) potential, including interactions between two backbone beads (AA), two side-chain beads (BB), and between the backbone and side-chain beads (AB). The effective diameters of beads A and B are captured through the LJ parameters: ${\sigma }_{\text{AA}}=5.5{\text{\AA}},\,{\sigma }_{{BB}}=4.42\,{\text{\AA}},\,{and}\,{\sigma }_{{AB}}=4.96{\text{\AA}}$, reflecting the different sizes of the backbone and side-chain groups. The corresponding masses are 85.1 g/mol for bead A and 15 g/mol for bead B, based on their chemical composition. Additionally, bonded interactions in the CG model include AA and AB bonds, AAA and AAB angles, and AAAA and BAAB dihedrals. Additional details on the parameterization of both bonded and non-bonded interactions can be found in previous studies^4,73,74^.

We use an in-house code to construct a centered nanoparticle with smooth, corrugated, and porous configurations, as shown in Fig. 1b–d, respectively. A simple cubic lattice structure is used to construct nanoparticle geometries, with beads evenly spaced at a distance of 3 $\mathring{\rm A}$ in all three directions. The smooth nanoparticle has a spherical shape. The corrugated nanoparticle is created by carving 14 evenly distributed holes, each approximately 3.5 nm in width and 1 nm in depth, from the smooth one. The porous nanoparticle is constructed by creating three through-holes with a diameter of 2.5 nm within the smooth one. This configuration results in a porosity ratio of 0.28, which is defined as the ratio of the mass of the porous nanoparticle to that of the smooth nanoparticle. All three types of nanoparticles have the same outer diameter of 9 nm, which is defined as the maximum distance between two beads on the surfaces of the nanoparticles. The weight fractions of nanoparticles in all three types of nanocomposites are kept the same at 0.25, resulting in different overall box sizes. In this study, we treat the nanoparticles in the coarse-grained models as rigid bodies to focus on examining the enhancement of mechanical properties of polymer matrices resulting from the nanoconfinement effect on polymer chains. This simplification increases computational efficiency while still effectively capturing the key characteristics of the nanocomposite systems, given that nanoparticles are often much stiffer than polymer matrices.

The interactions between PMMA beads and nanoparticle beads are modeled consistently with the LJ potential form^75^, as expressed in Eq. (2):

2 $$\begin{array}{cc}{E}_{{LJ}}\left(r\right)=4\varepsilon \left[{\left(\frac{\sigma }{r}\right)}^{12}-{\left(\frac{\sigma }{r}\right)}^{6}\right] & {\rm{for}}\,r < {r}_{c}\end{array}$$

where $r$ is the distance between a PMMA bead, either type A or type B, and a nanoparticle bead; $\varepsilon$ is the depth of the potential well, positively related to the strength of the interaction described by this potential; $\sigma$ corresponds to the equilibrium distance as $1.12\sigma$ is at which the potential between the two beads reaches its minimum.

The LJ parameter $\sigma$ governs the equilibrium distance between PMMA and nanoparticle beads. Experimental measurements and atomistic simulations suggest that the typical equilibrium distance between polymer chains and nanoparticle surfaces ranges from 2 to 5 Å^76–78^. To remain within this experimentally supported range while ensuring computational stability, we selected a representative value of 4.5 Å. To simulate a range of polymer-nanoparticle interfacial interactions, we use three values for the LJ energy parameter $\varepsilon$: 0.2, 1, or 5 ${{\rm{kcal}}}/{{\rm{mol}}}$, corresponding to weak, moderate, and strong adhesion, respectively. These values allow us to capture a wide spectrum of interfacial bonding scenarios. To benchmark the interfacial interactions in our simulations, we calculate the interfacial energy between PMMA and smooth nanoparticles for $\varepsilon =1\,{{\rm{kcal}}}/{{\rm{mol}}}$, which yields a value of approximately 0.14 ${\rm{J}}/{{\rm{m}}}^{2}$. This value is consistent with experimentally reported interfacial energies for typical polymer-nanoparticle systems^79,80^, supporting the physical relevance of our parameter selection.

### CG-MD simulation procedures

We generate the initial structures of different nanocomposite systems using in-house codes that prevent initial overlap between PMMA chains and nanoparticles. Following this, we perform simulations to fully equilibrate these nanocomposite systems before characterizing their mechanical properties with respect to nanoparticle configurations. All the simulations are carried out using the Large-scale Atomic/Molecular Massively Parallel Simulator (*LAMMPS*)^81^, while *OVITO* is used to visualize the simulation results^82^.

Initially, the model systems undergo energy minimization followed by equilibration under an NVE ensemble with limited bead movement (NVE/limit) to correct improper chain configurations^81,83^. During this stage, the maximum distance per bead in one timestep is restricted to 0.2 $\mathring{\rm A}$. To prevent excessively high energies at the start of the equilibration, we employ a soft potential^81^ with a cutoff of 0.1 $\mathring{\rm A}$ for the nonbonded interactions among PMMA beads, and a very low $\varepsilon$ (0.001 ${{\rm{kcal}}}/{{\rm{mol}}}$) is temporarily applied in the LJ potential between PMMA and nanoparticle beads. These settings are maintained for 50,000 timesteps. Subsequently, the actual non-bonded interactions for PMMA^73^ and for PMMA-nanoparticle interactions are reinstated, and the energy minimization and equilibration process is repeated for another 50,000 timesteps. The simulation ensemble is then switched from NVE/limit to NPT^84^. To quickly eliminate unrealistic voids, we subject the nanocomposite systems to ultra-high temperature and pressure. Since the CG force field is non-reactive (no bond or angle breaking occurs), this procedure effectively closes voids and facilitates the penetration of polymer chains through the porous nanoparticles without producing unphysical configurations. Next, the temperature and pressure are reduced to 300 K and 0 atm over 50,000 timesteps, followed by a 30,000-timestep hold. An annealing process then ensues: the temperature is raised to 600 K over 50,000 timesteps, held for 30,000 timesteps, reduced back to 300 K over 50,000 timesteps, and finally maintained at 300 K for an additional 30,000 timesteps. This annealing further equilibrates the system and alleviates residual stress. The resulting simulation trajectories confirm that, after equilibration, the polymer chains closely surround the nanoparticles with no visible voids, as well as penetrating the through-holes of porous nanoparticles.

After thorough equilibration, we characterize the thermodynamic properties of the nanocomposites depending on nanoparticle configurations. Specifically, we compute the molecular stiffnesses based on $\langle {u}^{2}\rangle$ in the caging regime, where particles are confined by their neighbors. In this regime, the molecular stiffness is inversely proportional to $\langle {u}^{2}\rangle$^85,86^. In our case, $\langle {u}^{2}\rangle$ is obtained from the mean square displacement (MSD) $\langle {r(t)}^{2}\rangle$ of the particles, measured at the onset of caging^53^. In our analysis, we evaluate both the mean-field molecular stiffness, computed as the average MSD of all PMMA beads, and the local molecular stiffness, determined from the MSD of PMMA beads within each small cell. This approach enables us to assess both the overall and region-specific effects of nanoparticles on the thermomechanical properties of the polymer matrix.

### Characterizing mechanical and viscoelastic properties

Additionally, we apply various loading conditions to the nanocomposite systems to explicitly quantify the reinforcement effects of the nanoparticles. In these nonequilibrium simulations, the temperature is maintained at 300 K. During uniaxial tensile tests, a strain rate of $5\times {10}^{-7}/{{\rm{fs}}}$ is used to stretch the cubic box along the *x*-direction, while the pressure in the other two directions is held at 0 atm. The stress generated in the polymer phase along the stretching direction is recorded to generate stress-strain curves. For shear tests, a shear strain rate of ${10}^{-6}/{{\rm{fs}}}$ is applied along the *xz*-plane, with the pressure normal to the shear plane maintained at 0 atm. We record the shear stresses along the shear plane for analysis. We note that the high strain rates are consistent with those employed in previous molecular dynamics studies, including our own prior work. While we acknowledge that strain rate can influence the absolute values of mechanical properties due to rate-dependent viscoelasticity, such limitations are inherent to molecular simulations. Nonetheless, we believe the key trends observed in our study—particularly the effects of nanoparticle surface morphology on stiffness and damping—are robust and would remain qualitatively valid under slower, more experimentally relevant strain rates.

We also perform small amplitude oscillatory shear (SAOS) tests^87^ to investigate the viscoelastic properties of the nanocomposites. Specifically, the nanocomposite systems are subjected to a time-dependent oscillatory shear strain expressed in Eq. (3):

3 $$\gamma \left(t\right)={\gamma }_{0}\sin (\omega t)$$

where ${\gamma }_{0}$ is the amplitude of the oscillatory shear strain, $\omega$ is the angular frequency, and $t$ is the time in ${ps}$. In this study, we set ${\gamma }_{0}=0.05{\rm{rad}}$ and $\omega =4\pi \times {10}^{-2}{{\rm{rad}}}/{{\rm{ps}}}$, consistent with previous studies^74,87^.

The stress response from the SAOS tests is expected to follow:

4 $$\sigma \left(t\right)={\sigma }_{0}\sin (\omega t+\delta )$$

where ${\sigma }_{0}$ is the amplitude of the oscillatory stress, $\omega$ is the angular frequency equal to that of the applied shear strain, and $\delta$ is the phase shift relative to the input strain.

The storage modulus ($G^{\prime}$) and loss modulus ($G^{{\prime} {\prime}}$) are then calculated as:

5 $$G^{\prime} =\frac{{\sigma }_{0}}{{\gamma }_{0}}\cos (\delta )$$

6 $${G}^{{\prime} {\prime} }=\frac{{\sigma }_{0}}{{\gamma }_{0}}\sin (\delta )$$

and the loss tangent is given by:

7 $$\tan \left(\delta \right)=\frac{{G}^{{\prime} {\prime} }}{{G}^{{\prime} }}$$

The reported results of Young’s modulus, shear modulus, $G^{\prime}$, $G^{{\prime} {\prime}}$, and $\tan \left(\delta \right)$ include standard deviations obtained from five independent simulations with different initial configurations, providing a measure of reproducibility and statistical reliability in our results.

### Calculation of the local shear strain

To calculate the local shear strain ${\varepsilon }_{{xz\_l}}$, the simulation box is divided into $19\times 19\times 19$ equal-sized cells. Within each cell, we compute the average displacement in the x-direction, denoted as ${u}_{x}^{(i,j,k)}$, where *i*, *j*, *k* = 1, 2, …, 19 correspond to the cell indices along the x-, y-, and z-directions, respectively.

The local shear strain at the center of each cell is calculated as:

8 $${\varepsilon }_{{xz}{{\_}}l}^{(i,j,k)}=\left\{\begin{array}{cc}\left.\left({u}_{x}^{\left(i,j,k+1\right)}-{u}_{x}^{\left(i,j,k\right)}\right)\right/{L}_{z}^{(c)} & k=1\\ \left.\left({u}_{x}^{\left(i,j,k+1\right)}-{u}_{x}^{\left(i,j,k-1\right)}\right)\right/\left({2L}_{z}^{\left(c\right)}\right) & 1 < k < 19\\ \left.\left({u}_{x}^{\left(i,j,k\right)}-{u}_{x}^{\left(i,j,k-1\right)}\right)\right/{L}_{z}^{(c)} & k=19\end{array}\right.$$

where ${L}_{z}^{(c)}$ is the cell size in the z-direction. For simplicity and computational feasibility, displacements from periodic images are not considered when evaluating cells near the lower and upper z-boundaries. However, our analysis focuses on the interior cells surrounding the central nanoparticle, where boundary effects are negligible and local variations are most representative.

To examine the plane at $y=0$ in Fig. 5, we extract the cells with $j=10$ and calculate the shear strain at the center of each cell. Shear strain values at arbitrary locations within this plane are obtained through polynomial interpolation based on the cell-centered values and subsequently smoothed to enhance visual clarity.

## Data Availability

The authors confirm that the data supporting the findings of this study are available within the article. Additional data and simulation codes are available from the corresponding author, Z.M., upon reasonable request.
